# Expression Patterns of Escape Genes in Turner Syndrome Fibroblasts and Induced Pluripotent Stem Cells

**DOI:** 10.3390/ijms26030975

**Published:** 2025-01-24

**Authors:** Seki Byun, Sang-Hoon Yoon, Yean-Ju Hong, Hyun-Sik Jang, Bong-Jong Seo, Gyu-Tae Choi, Hyeonwoo La, Je-Woo Lee, Kwonho Hong, Jeong-Tae Do

**Affiliations:** Department of Stem Cell and Regenerative Biotechnology, Konkuk Institute of Technology, Konkuk University, 120 Neungdong-ro, Gwangjin-gu, Seoul 133-702, Republic of Korea; alvna@konkuk.ac.kr (S.B.); yun.sanghun@cira.kyoto-u.ac.jp (S.-H.Y.); ndhong7@gmail.com (Y.-J.H.); huyndig@naver.com (H.-S.J.); sbj1990@naver.com (B.-J.S.); ch08david@naver.com (G.-T.C.); lahw94@gmail.com (H.L.); wpdn1541@naver.com (J.-W.L.); hongk@konkuk.ac.kr (K.H.)

**Keywords:** pluripotent stem cells, turner syndrome, reprogramming, escape gene, X-chromosome inactivation

## Abstract

Turner syndrome (TS) is an X monosomy-related disorder caused by X chromosome nondisjunction during embryonic development. Patients with TS have only one intact X chromosome, with the other either completely or partially lost. TS affects various tissues, including the liver, kidneys, brain, cardiovascular system, and ovaries. These abnormalities are suggested to involve an altered dosage of escape genes that evade X chromosome inactivation. However, the mechanisms and roles of these escape genes in the TS phenotype remain unclear. We hypothesized that the expression levels of escape genes differ between wild-type (WT) and TS cell lines. In this study, we generated induced pluripotent stem cell (iPSC) lines from WT and TS fibroblasts and examined the expression levels of escape genes in both undifferentiated fibroblasts and reprogrammed iPSCs from WT and TS samples. The reprogrammed WT and TS iPSCs exhibited general characteristics of pluripotency, including the expression of pluripotency markers and the potential to differentiate into all three germ layers. Forty-five escape genes were differentially expressed between the WT and TS cell lines. Among these, five genes (*ATP7A*, *PHKA1*, *EBP*, *ZFX*, and *SMC1A*) were suggested to be implicated in the TS phenotype. However, further studies using additional cell lines are necessary to clarify the correlation between TS and escape genes.

## 1. Introduction

Turner syndrome (TS) is a disorder occurring mainly in females owing to the complete or partial loss of one X chromosome. Approximately half of TS patients exhibit X monosomy (45,X), while others are affected by deletions, structural defects [1,2], and mosaicism of one X chromosome [3,4]. TS patients typically present with short stature, webbed neck, cubitus valgus, gonadal failure, and congenital cardiovascular and skeletal anomalies [5,6,7]. TS is one of the most common forms of X chromosome monosomy, with a prevalence of less than 3% in developing embryos and occurring in approximately 1 in 1500 to 2500 live female births [8,9]. Most embryos with X monosomy do not survive pregnancy, with fewer than 1% of 45,X embryos developing to term, and only 3.8% of TS patients have live-born children [10,11,12]. However, the precise mechanisms underlying the TS phenotype remain poorly understood. According to Bondy et al., embryos with a complete 45,X karyotype do not survive, and TS patients who are born typically have cryptic mosaicism, such as 45,X/46,XX or 45,X/46,XY, present in their cells [13].

In normal females who have two X chromosomes (46,XX), one of the two X chromosomes undergoes chromosome-wide inactivation during early development, a process known as X chromosome inactivation (XCI), resulting in only one active X chromosome (Xa) [14]. Once established, the inactive state of the inactive X chromosome (Xi) is maintained throughout life, except in the germ cell lineage [15]. Although most genes on the Xi are silenced, some genes escape this inactivation and are expressed from both the Xa and Xi; these are known as “escape genes” [16]. In humans, approximately 15% of X-linked genes on the Xi escape XCI [16].

We hypothesized that the TS phenotype may be influenced by the lack of expression of escape genes on the Xi [17,18,19] and that differences in the expression levels of escape genes may exist between wild-type (WT) and TS cell lines. To investigate this, we aimed to verify the differences in gene expression patterns of escape genes between TS and normal female cells (fibroblasts and pluripotent stem cells). We reprogrammed TS fibroblast cells into induced pluripotent stem cells (iPSCs) and monitored changes in escape gene expression patterns before and after the reprogramming process [20,21,22].

## 2. Results

### 2.1. Generation of Integration-Free TS iPSCs

**Figure 1:**
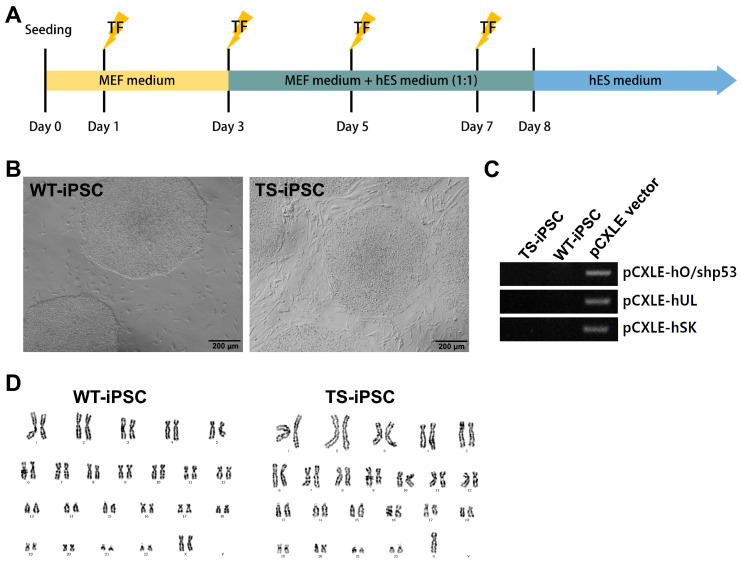
Establishment of Turner syndrome (TS) iPSCs and WT-iPSCs. (**A**) Schematic diagram of the integration-free episomal reprogramming protocol. (**B**) iPSCs established from both WT and TS fibroblasts exhibited morphology similar to human embryonic stem cell colonies. (**C**) Genotyping confirmed that the iPSC lines were free of reprogramming vectors, using the reprogramming plasmid as a positive control. (**D**) Karyotype analysis showed that WT-iPSCs had a normal female karyotype, while TS-iPSCs displayed typical TS karyotypes (45,X).

To generate pluripotent stem cells from the somatic cells of a TS patient, we derived induced pluripotent stem cells (iPSCs) from fibroblasts using episomal vectors. Both TS and female WT fibroblasts were reprogrammed using pCXLE-based episomal vectors containing OCT4, KLF4, SOX2, L-MYC, Lin28, and shRNA-P53. The reprogramming vectors were transfected on days 1, 3, 5, and 7 (Figure 1A). ESC-like colonies appeared around 14 days after transfection and were manually picked and passaged on feeder layers every 7 days. No morphological differences were observed between WT and TS iPSCs (Figure 1B).

To confirm the absence of reprogramming vector integration in the iPSC lines, we performed genotyping for the pCXLE vector. The analysis indicated that both TS and WT iPSCs were generated without integration of the pCXLE vector (Figure 1C). Karyotype analysis revealed that WT iPSCs were 46,XX and TS iPSCs were 45,X, confirming that TS iPSCs were derived from TS somatic cells and maintained X chromosome monosomy during reprogramming (Figure 1D).

### 2.2. Characterization of TS-iPSCs

**Figure 2:**
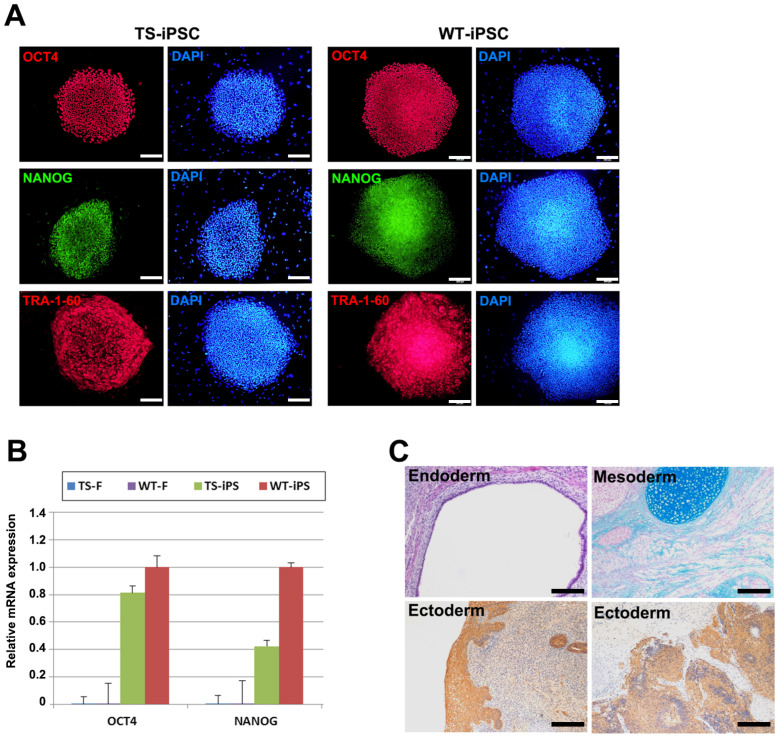
Characterization of pluripotency in WT and TS-iPSCs. (**A**) Immunofluorescence images of WT and TS-iPSCs stained with pluripotent markers (OCT4, NANOG, and TRA-1-60). Scale bar = 200 µm. (**B**) Quantitative reverse transcription polymerase chain reaction (qRT-PCR) analysis of pluripotency marker genes (*OCT4* and *NANOG*) in WT and TS fibroblasts and WT and TS-iPSCs. (**C**) Teratoma formation assay using TS-iPSCs, demonstrating contribution to all three germ layers after 8 weeks of injection into immunodeficient mice. Endodermal, ectodermal, and mesodermal tissues were stained with hematoxylin/eosin, PAS, and Alcian blue, respectively. Magnification ×100.

**Figure 3:**
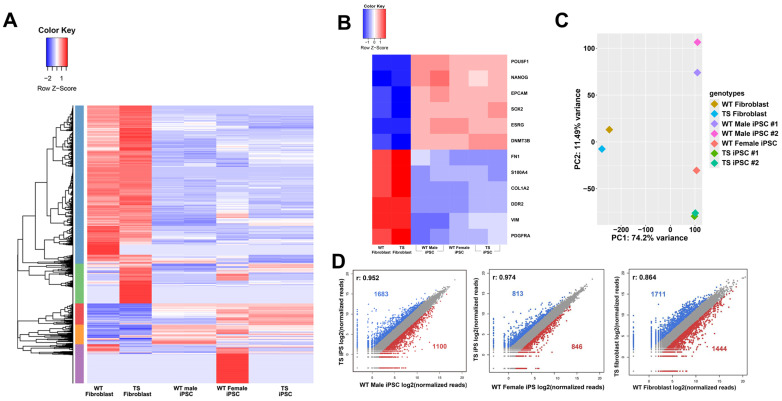
RNA sequencing transcriptome analysis of fibroblasts and iPSCs. (**A**) Heat map of two-way hierarchical clustering using Z-scores for normalized values (log2 based). (**B**) Heat map showing expression levels of pluripotency and fibroblast markers. (**C**) Principal component analysis (PCA) plot of gene expression in TS fibroblasts, TS-iPSCs, WT fibroblasts, and WT-iPSCs. (**D**) Pairwise comparison of expression levels between TS and WT cell lines.

To confirm the pluripotency of the established iPSC lines, we investigated the expression of pluripotency markers and the differentiation potential of the three germ layers. Immunofluorescence analysis showed that both TS and WT iPSCs expressed pluripotency markers, including OCT4, NANOG, and TRA-1-60 (Figure 2A). Quantitative real-time RT-PCR (qRT-PCR) analysis also showed upregulated levels of general pluripotency markers *OCT4* and *NANOG* in both WT and TS iPSCs (Figure 2B). RNA sequencing analysis further confirmed the upregulation of pluripotency markers such as *OCT4*, *NANOG*, *EPCAM*, *SOX2*, *ESRG*, and *DNMT3B*, along with the downregulation of tissue-specific markers such as *FN1* (*FIBRONECTIN1*), *S100A4*, *COL1A2*, *DDR2*, *VIM*, and *PDGFRA* (Figure 3B).

The differentiation potential was confirmed by a teratoma formation assay. Undifferentiated TS iPSCs were mixed with Matrigel and injected into the testis capsule of immunodeficient mice. Eight weeks post-engraftment, teratomas were recovered, fixed, and subjected to histological analysis. The histological examination revealed that the teratomas contained tissues from all three germ layers, including endoderm, mesoderm, and ectoderm tissues (Figure 2C).

### 2.3. Global Gene Expression Patterns of Fibroblasts and iPSCs

RNA sequencing analysis was conducted to compare the transcriptomes of TS fibroblasts, WT fibroblasts, TS iPSCs, and WT iPSCs. Heat maps and pair-wise comparison analyses demonstrated substantial differences between iPSCs and fibroblasts (Figure 3A, Supplementary Figure S1A). Pluripotency markers were upregulated and fibroblast markers were downregulated in both TS and WT iPSCs (Figure 3B). Notably, although TS fibroblasts exhibited significant differences compared to WT fibroblasts, gene expression patterns of WT iPSCs were found to be more similar to TS iPSCs (Figure 3C,D), suggesting that the reprogramming process could reduce the gap in gene expression patterns between WT and TS fibroblasts.

**Figure 4:**
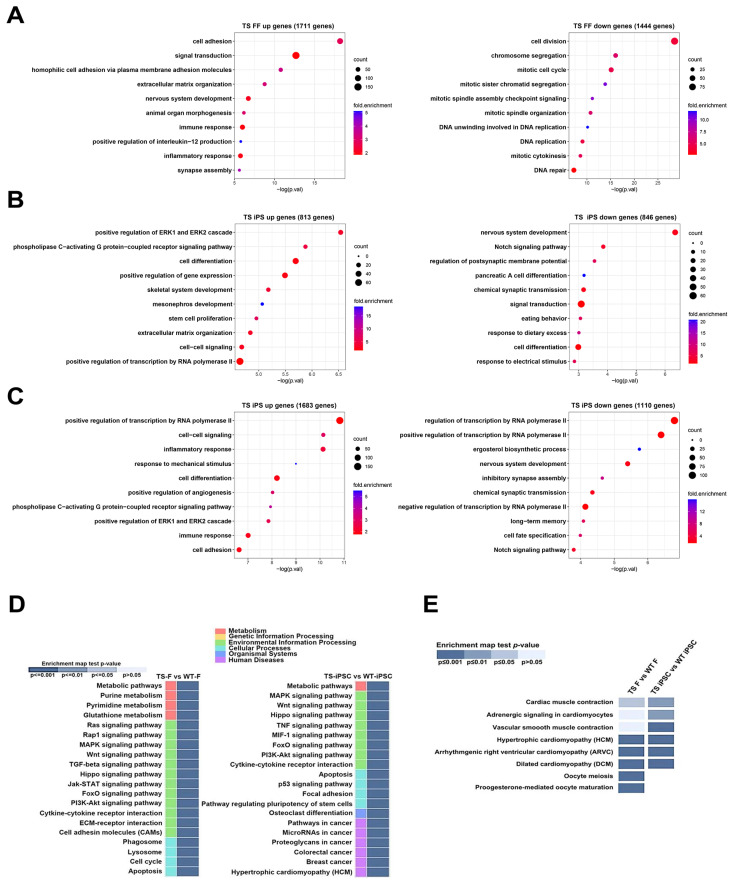
GO and KEGG pathway analysis of DEGs. Gene-Enrichment and Functional Annotation Analysis using Gene Ontology in the Biological Process category, showing the top 10 enriched GO terms (up- and down-regulated genes) among differentially expressed genes between WT- and TS fibroblasts (**A**), WT female and TS-iPSCs (**B**), and WT male and TS-iPSCs (**C**). (**D**) Clustered heat map of KEGG pathway enrichment analysis, showing pathways in fibroblast and iPSC lines. Color intensities indicate the enrichment score of each KEGG pathway. (**E**) Selected KEGG pathways potentially involved in Turner syndrome phenotypes.

Gene ontology (GO) analysis was conducted to categorize genes based on their functions in both TS and WT cell lines. Initially, we compared differentially expressed genes in biological processes between WT and TS fibroblasts before reprogramming. In TS fibroblasts, genes related to cell adhesion and the regulation of cellular interactions and signaling processes were upregulated, while genes related to cellular division and replication were downregulated (Figure 4A). This suggested an increase in cellular interactions, immune responses, and tissue development, possibly associated with inflammation or immune-related processes, and a decrease in cellular proliferation and division.

The analysis was then conducted on iPSCs from both TS and WT female iPSC lines. TS iPSCs exhibited the upregulated expression of genes associated with various cellular and developmental processes, including cell specialization, signal transduction, organ development, extracellular structure organization, intercellular communication, and the regulation of gene expression and cell proliferation, compared to WT female iPSCs (Figure 4B). In contrast, TS iPSCs showed the downregulation of genes associated with neuronal development, cellular communication in the nervous system, genetic regulation, organ formation, and responses to external stimuli such as dietary conditions compared to WT female iPSCs (Figure 4B). This suggests that TS iPSCs may experience deficiencies in tissue development, compromised cellular communication, and disruptions in regulatory mechanisms governing gene expression and cell growth.

Lastly, subsequent analysis was conducted on iPSCs derived from TS and WT male iPSC cell lines. WT male iPSCs were chosen because they have only one X chromosome like TS iPSCs, although they also contain a Y chromosome. TS iPSCs displayed the upregulated expression of genes associated with processes critical for cellular communication, immune response, and tissue remodeling. On the other hand, TS iPSCs showed the downregulation of genes involved in neuronal development and differentiation, including nervous system development, inhibitory synapse assembly, and chemical synaptic transmission. Furthermore, the downregulation of transcriptional regulators, such as RNA polymerase II and the Notch signaling pathway, suggested impaired gene expression and disrupted developmental signaling. Collectively, these results highlight limitations in neural development and differentiation in TS iPSCs, accompanied by deficiencies in transcriptional control mechanisms (Figure 4C).

KEGG pathway analysis revealed that differentially expressed genes between TS and WT fibroblasts were enriched for terms associated with ‘Metabolic pathways’, ‘Cell cycle’, ‘Pathways in cancer’, ‘HTLV-I infection’, ‘Human papillomavirus infection’, and ‘DNA replication’. The differentially expressed genes between TS and WT iPSCs were enriched for terms associated with ‘HTLV-I infection’, ‘PI3K-Akt signaling pathway’, ‘MAPK signaling pathway’, ‘Cytokine-cytokine receptor interaction’, and ‘Breast cancer’ (Figure 4D). Intriguingly, genes involved in cardiomyopathy, a common symptom of TS, were differentially expressed between TS and WT cell lines (both fibroblasts and iPSCs). Additionally, genes important for oocyte generation were differentially expressed between TS and WT fibroblasts (Figure 4E).

### 2.4. Differentially Expressed X-Linked Escape Genes from Fibroblasts and iPSCs

**Figure 5:**
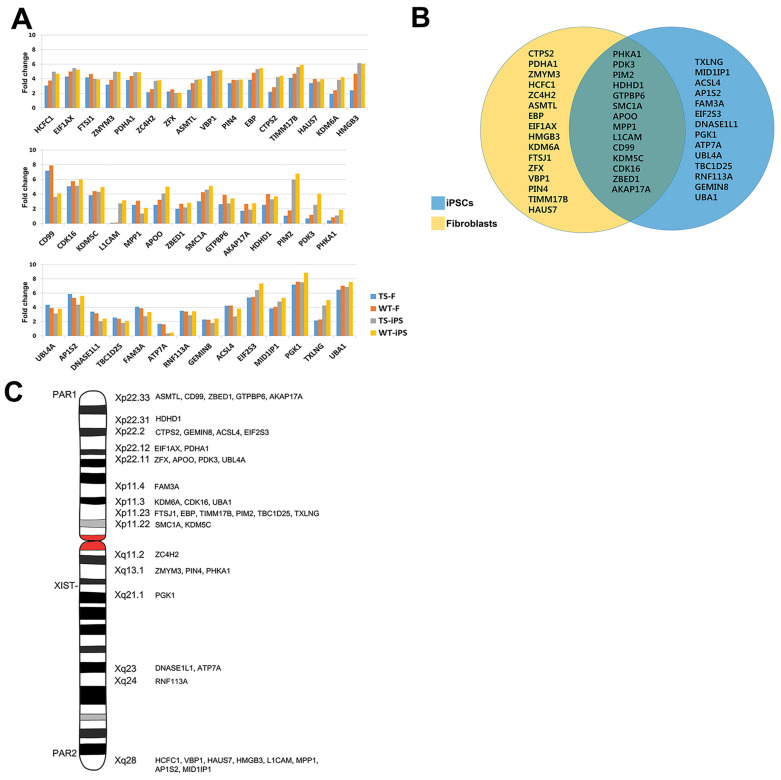
Expression patterns of X-linked genes and escape genes. (**A**) Forty-four escape genes sorted according to the criterion for escape gene expression in fibroblast and iPSC lines. (**B**) Differential expression of escape genes between WT and TS fibroblasts (yellow circle) and WT and TS-iPSCs (blue circle). The overlapping area of the two circles represents escape genes differentially expressed in both fibroblasts and iPSCs. (**C**) Illustration showing the locations of the 44 genes on the X chromosome.

To determine whether these expression differences were caused by X chromosome monosomy, we compared X-linked genes between cell lines. Considering the expression of escape genes in Xi, we expected the expression level of escape genes from both Xa and Xi to be 1.1- to 2.0-fold higher in normal cell lines than in TS cell lines. First, we identified 119 escape genes from the X-linked genes. These genes were then further sorted according to the criterion for escape gene expression (Figure 5A, Supplementary Figure S2A). Since the expression pattern of escape genes appears in a tissue-specific manner, we classified the escape genes according to cell types. Forty-four genes analyzed were primarily located within or near the PAR1 and PAR2 regions, where many escape genes are found (Figure 5C). Of the 44 genes, 30 were more highly expressed in WT fibroblasts compared to TS fibroblasts (yellow circle in Figure 5B), 28 were more highly expressed in WT female iPSCs compared to TS iPSCs (blue circle in Figure 5B), and 14 genes were more highly expressed in WT cells compared to TS cells (overlapping area of yellow and blue circle in Figure 5B). Among the 44 genes, the expression level of 6 genes (*PIN4*, *KDM6A*, *MPP1, HDHD1*, *PHKA1*, and *RNF113A*) was higher in WT male iPSCs compared to TS iPSCs (green circle in Supplementary Figure S2B), 33 were more highly expressed in WT female iPSCs compared to TS iPSCs (pink circle in Supplementary Figure S2B), and 5 genes were more highly expressed in both WT iPS cell types compared to TS iPS cells (overlapping area of green and pink circle in Figure S2B).

## 3. Discussion

Although many studies have been conducted on TS, the specific genes involved in the TS phenotype remain largely uncharacterized [23]. The most evident difference between TS patients and normal women is that TS patients have only one intact X chromosome. Due to XCI, one intact X chromosome appears to be sufficient, but not all genes on the inactive X chromosome (Xi) are silenced. Some genes escape XCI and contribute to overall gene expression levels [4,24]. Therefore, we aimed to identify the relationship between the TS phenotype and escape genes. We compared the expression patterns between TS cell lines (fibroblasts and iPSCs) and normal female cell lines (fibroblasts and iPSCs) [25]. We found that 44 escape genes were differentially expressed between WT and TS cell lines. Of these, 30 escape genes in TS fibroblasts and 28 escape genes in TS iPSCs were less expressed than their counterparts. Only 14 genes were consistently less expressed in both TS fibroblasts and TS iPSCs compared with WT fibroblasts and WT iPSCs. The number of escape genes differentially expressed between TS and WT iPSCs was approximately half that observed between TS and WT fibroblasts. One possible explanation is the reactivation of the Xi chromosome in TS fibroblasts after reprogramming and the acquisition of pluripotency. The expression levels of XIST, which is expressed from Xi, were high in WT fibroblasts but almost absent in TS fibroblasts, as TS fibroblasts have only one X chromosome (Supplementary Figure S1B). After reprogramming, the highly expressed level of XIST in WT fibroblasts was downregulated, suggesting that Xi in WT fibroblasts might be reactivated (Supplementary Figure S1B). Therefore, the number of active X chromosomes (Xa) changes after reprogramming: both WT and TS fibroblasts initially have one Xa, but WT iPSCs have two Xa, while TS iPSCs continue to have one Xa after reprogramming.

We also found that some escape genes differentially expressed between the TS and WT cell lines might be involved in cardiovascular disease, mental retardation, and ovarian development. *ATP7A* and *PHKA1* are likely related to the cardiovascular system [26,27], *EBP* is associated with coronary heart disease and skeletal-related conditions [28], *ZFX* is known to be involved in animal size and ovarian failure [29], and *SMC1A* is implicated in premature ovarian failure [30]. Given that some escape genes were differentially expressed between WT and TS cells, we speculate that these escape genes might contribute to cardiac disease and ovarian failure in TS.

Little is known about the spatiotemporal mechanism of escaping from XCI. Escape genes appear to be silenced in the initial stages of development and reactivated later [31]. Thus, we expected that the level of escape genes in undifferentiated iPSCs might not differ significantly between WT and TS cell lines. However, we found that 30 escape genes were less expressed in TS fibroblasts compared with WT fibroblasts. We hypothesize that different escape genes may be differentially expressed in various tissue types of TS patients, as TS affects many tissues, including the liver, kidneys, brain, cardiovascular system, and ovaries [4]. Therefore, further studies on the escape genes related to TS phenotypes must be conducted in various tissues and cell types to identify which escape genes are involved in specific tissues. Experiments using more cell lines might offer a better understanding of the correlation between TS and escape genes.

## 4. Materials and Methods

### 4.1. Cell Culture and iPSC Generation

The TS fibroblasts used in this study, originating from a female sample, were the GM01176 line from the Coriell Institute for Medical Research (Camden, NJ, USA). Normal female fibroblasts, SMC-FF (WT fibroblasts), were derived from Samsung Medical Center (Seoul, Republic of Korea). The male iPSCs, CMC-011 (WT male iPSC), originated from the National Stem Cell and Regenerative Center (Cheongju, Republic of Korea). All fibroblasts were cultured in Dulbecco’s Modified Eagle’s Medium (DMEM; Gibco, Grand Island, NY, USA), supplemented with 15% fetal bovine serum (FBS; Gibco), 1× penicillin/streptomycin/glutamine, 1 mM nonessential amino acids (NEAA; Gibco), and 0.1 mM β-mercaptoethanol (Gibco). Human dermal fibroblasts (HDFs) were transduced with reprogramming factor sequences using episomal vectors (OCT4, KLF4, SOX2, L-MYC, Lin28, and small hairpin RNA for p53). A total of 3 µg of expression plasmid mixtures was transfected into 1 × 10^5^ HDFs using Lipofectamine 2000 (Invitrogen, Waltham, MA, USA) on days 1, 3, and 5 post-seeding.

The following day, the medium was switched to Human ESC Medium [DMEM/F12 (Gibco) with 20% KnockOut Serum Replacement (KoSR; Gibco), 1× penicillin/streptomycin/glutamine, 1 mM NEAA (Gibco), 0.1 mM β-mercaptoethanol (Gibco), and 10 ng/mL basic FGF (FGF2; Invitrogen)]. The medium was refreshed every other day. iPSC colonies resembling human ESCs were selected 30 days post-transfection for further cultivation and evaluation. The selected iPSCs were maintained on feeder cells (CF1, mouse embryonic fibroblasts) in HES medium at 37 °C with 5% CO_2_.

For the feeder-free culturing of iPSCs, the plates were coated with Vitronectin (Gibco). The cells were then divided into clumps, cultured in Tesr-e8 medium (Stemcell, Vancouver, BC, Canada), and cryopreserved every five passages. The iPSCs created and cells purchased were used for analysis at fewer than 30 passages.

### 4.2. Karyotype Analysis

Metaphase chromosomes were arrested by adding colcemid (Gibco, 10 µg/mL). The cells were then subjected to hypotonic treatment (0.075 M KCl + 1% sodium citrate) and washed with a fixative (methanol/acetic acid = 3:1) multiple times. The cell pellet was spread on slides, air-dried, and then GTG-banded. The slides were examined under a light microscope at 1000× magnification.

### 4.3. RT-PCR Analysis

PCR premix was used for RT-PCR, and 100 ng of genomic DNA was used. The PCR protocol included 28 cycles of 30 s at 94 °C, 30 s at 60 °C, and 30 s at 72 °C. For integration checks, RT-PCR assays included human embryonic stem cells (hESCs) as negative controls and episomal plasmids as positive controls. The primers for qRT-PCR were as follows: pCXLE-ho/shp53 sense: 5′-CATTCAAACTGAGGTAAGGG-3′, antisense: 5′-TAGCGTAAAAGGAGCAACATAG-3′; pCXLE-hSK sense: 5′-TTTGTTTGACAGGAGCGACAAT-3′, antisense: 5′-TTCACATGTCCCAGCACTACCAGA-3′; pCXLE-hUL sense: 5′-AGCCATATGGTAGCCTCATGTCCGC-3′, antisense: 5′-TAGCGTAAAAGGAGCAACATAG-3′.

### 4.4. Immunocytochemistry Experiments

Cells were fixed with 4% paraformaldehyde for 20 min at room temperature. After washing with phosphate-buffered saline (PBS, Welgene, Gyeongsan-si, Republic of Korea), the cells were treated with PBS containing 10% normal goat serum and 0.03% Triton X-100 for 45 min at room temperature. The primary antibodies used were anti-Nestin (monoclonal, 1:500, Millipore, Burlington, MA, USA) and anti-Sox2 (polyclonal, 1:500, Millipore). Fluorescently labeled secondary antibodies (Alexa Fluor 488 or 568; Molecular Probes, Eugene, OR, USA) were used according to the manufacturer’s specifications.

### 4.5. RNA Isolation and Real-Time RT-PCR

Total RNA was isolated using the RNeasy Mini Kit (Qiagen, Hilden, Germany) following the manufacturer’s protocol. cDNA was synthesized from 1 µg of total RNA using SuperScript III reverse transcriptase (Invitrogen, NY, USA). GAPDH was used as a reference control for real-time RT-PCR. PCR efficiency was corrected using the Relative Quantification Software (https://diagnostics.roche.com/global/en/products/instruments/lightcycler-480-ins-445.html, accessed on 1 August 2024, LightCycler® 480 Instrument II, Roche, Basel, Switzerland). Standard curves for each gene were created using known quantities of total cDNA. Thermal cycling conditions included 40 cycles of 30 s at 94 °C, 30 s at 60 °C, and 30 s at 72 °C. The primers for qRT-PCR were as follows: OCT4 (endo) sense: 5′-GACAGGGGGAGGGGAGGAGCTAGG-3′, antisense: 5′-CTTCCCTCCAACCAGTTGCCCCAA-3′; NANOG (endo) sense: 5′-CGCCCTGCCTAGAAAAGACA-3′, antisense: 5′-AAGCAAAGCCTCCCAATCCC-3′; DNMT3B sense: 5′-GTGTGTAGCTTAGCAGACTGG-3′, antisense: 5′-AGGGAAGACTCGATCCTCGTC-3′; OTX2 sense: 5′-GACCACTTCGGGTATGGACT-3′, antisense: 5′-TGGACAAGGGATCTGACAGT-3′.

### 4.6. Teratoma Formation

Immunodeficient Balb/c Nude mice (5 weeks old, male) were purchased from Orient Bio (Gyeonggi-do, Republic of Korea). iPSCs were injected into the testis capsules of these mice. Teratomas were surgically harvested 8 weeks post-injection. The teratomas were fixed in 4% paraformaldehyde (Sigma, Darmstadt, Germany), processed through graded ethanol, embedded in paraffin, and stained with hematoxylin/eosin (endoderm), Alcian blue (mesoderm), and PAS (ectoderm). Immunohistochemistry for Nestin and Musashi was performed using anti-Nestin (monoclonal, 1:100; Millipore) and anti-Musashi (monoclonal, 1:50; Millipore) antibodies.

### 4.7. RNA Sequencing

Starting with 1000 ng of total RNA, polyadenylated RNA was purified using oligo-dT magnetic beads, fragmented, and converted into single-stranded cDNA using reverse transcriptase and random hexamer primers. Actinomycin D was added to inhibit DNA-dependent synthesis of the second strand. Double-stranded cDNA was created by removing the RNA template and synthesizing the second strand in the presence of dUTP. The cDNA was prepared for sequencing using the TruSeq Stranded mRNA Library Prep Kit (Illumina, 20020595, San Diego, CA, USA) as per the manufacturer’s instructions. The library underwent sequencing using Illumina Novaseq 6000.

### 4.8. Bioinformatics

For post-sequencing data analysis, adapter sequences in the raw reads were trimmed with Skewer [32] (v0.2.2) with the –L 100 and -e options specified. The trimmed reads were then mapped to the mm10 UCSC mouse reference genome using the STAR [33] (v2.7.10b) aligner. Read quantification was performed with VERSE [34] (v0.1.5) using the -S option. Sample normalization was conducted using the median of ratios method implemented in the DESeq2 [35] (v1.42.1) package within R (v4.3.0; R Core Team 2023).

To generate the heatmap, *p*-values for each gene were calculated using the aov function in R. Genes with a *p*-value less than 0.01 and a fold change greater than 2 between any two samples were selected. These genes were clustered using hierarchical clustering, grouped into five distinct clusters, and visualized with the heatmap2 function of the gplots [36] (v3.2.0) package. Expression levels of pluripotency and fibroblast markers were also visualized using the heatmap2 function. Principal component analysis (PCA) was performed using the prcomp function in R to calculate principal components for each sample, with visualization created using ggplot2 [37] (v3.5.1). For pairwise comparisons, differentially expressed genes (DEGs) were defined as those with normalized expression values greater than 3 and a fold change greater than 3 between the two samples. Gene ontology (GO) analysis of the resulting DEGs was carried out using DAVID [38] (v2024q2), and the results were visualized with ggplot2 [37] (v3.5.1). The X escape gene was referenced from Ahern et al. [39].

### 4.9. Ethical Approval

All animal experiments were conducted in accordance with the guidelines approved by the Institutional Animal Care and Use Committee (IACUC) of Konkuk University (approval no. KU23148). Additionally, any research involving human participants was reviewed and approved by the Institutional Review Board (IRB) of Konkuk University (approval no. 7001355-201804-LR-240).

## Data Availability

The data that support the findings of this study are available from the corresponding author upon reasonable request.

## Supplementary Materials

The following supporting information can be downloaded at https://www.mdpi.com/article/10.3390/ijms26030975/s1.
