# Supplementary material for: Expression Patterns of Escape Genes in Turner Syndrome Fibroblasts and Induced Pluripotent Stem Cells
Source: Int J Mol Sci. 2025 Jan 24;26(3):975. doi: 10.3390/ijms26030975 (PMC11816654; doi:10.3390/ijms26030975)
Supplement: Supplementary file 1 [file ijms-26-00975-s001.zip › ijms-3362315-supplementary.pdf]

Supplementary Materials

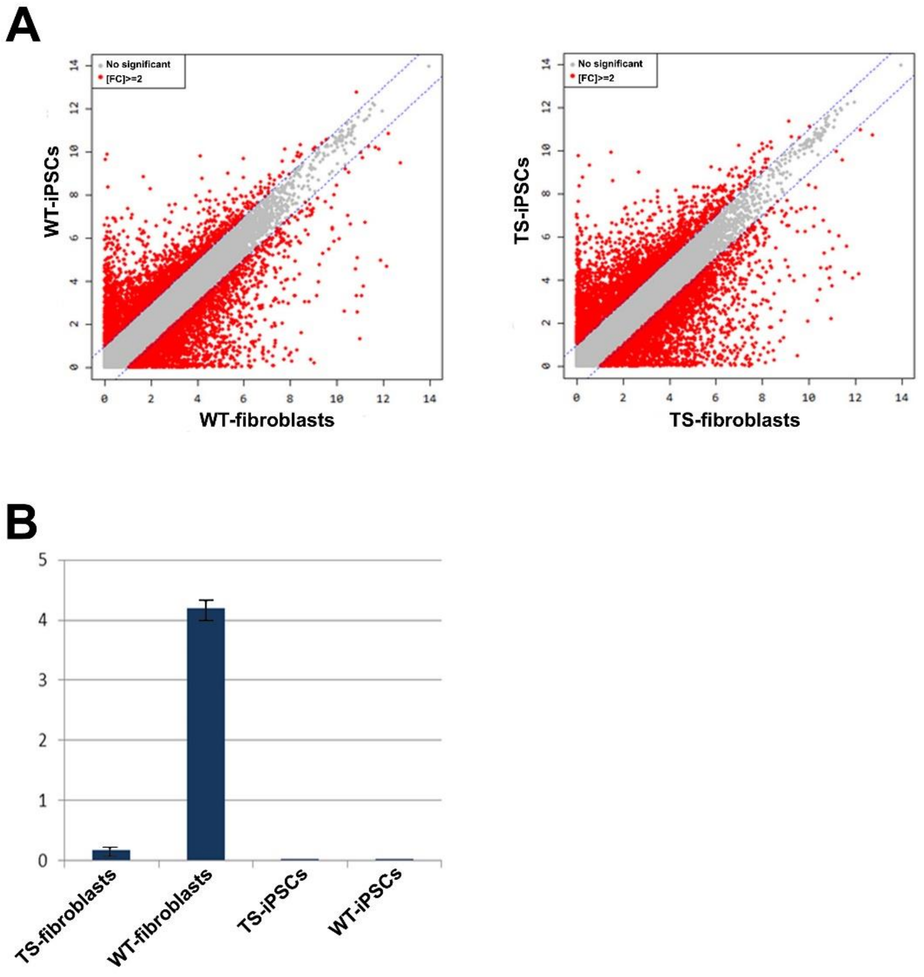

Figure S1. Gene expression patterns in fibroblasts and their pluripotent counterparts (WT and TS fibroblasts, and WT and TS-iPSCs). (**A**) Pairwise comparison of global gene expression in fibroblasts and reprogrammed iPSCs. (**B**) XIST expression levels in WT and TS fibroblasts, and WT and TS-iPSCs.

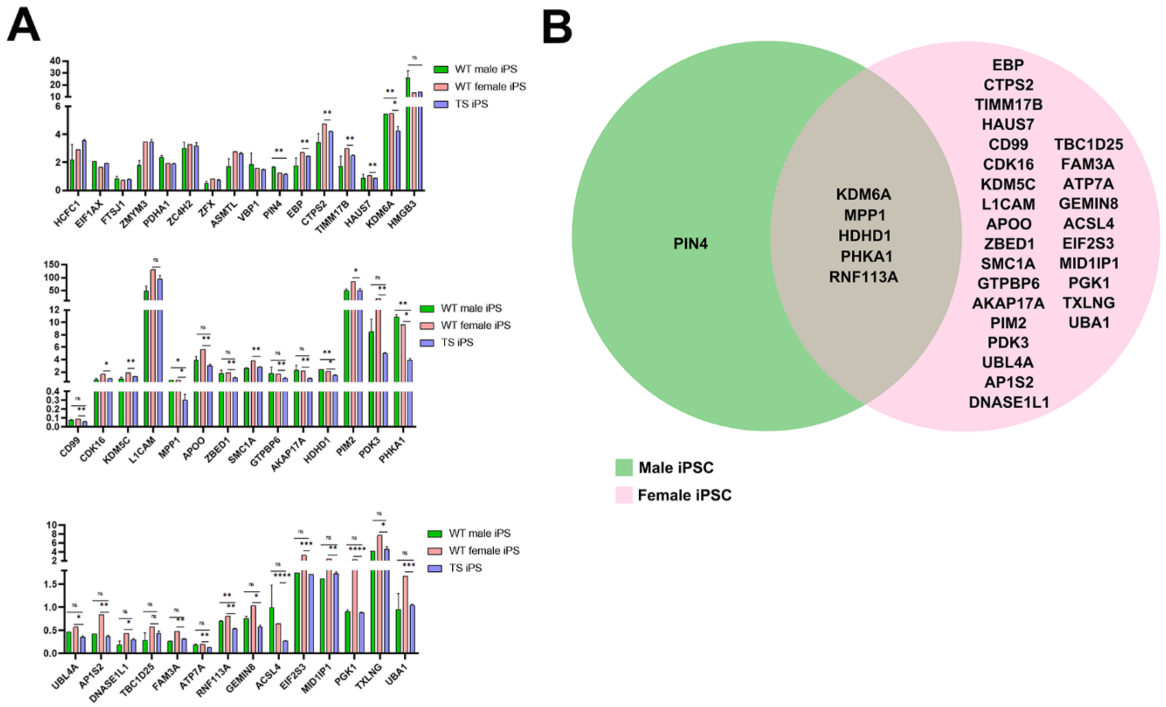

Figure S2. Expression patterns of X-linked genes in iPSCs (WT male, WT female and TS-iPSCs). (**A**) Expression criteria identified 44 escape genes in iPSC lines. (**B**) Escape genes differentially expressed between WT male and TS iPSCs (green circle) and WT female and TS iPSCs (pink circle). The shared region of the two circles indicates escape genes with differential expression in both WT male and WT female iPSCs compared with TS iPSCs.
